# Brain magnetic resonance imaging radiomics features associated with hepatic encephalopathy in adult cirrhotic patients

**DOI:** 10.1007/s00234-022-02949-2

**Published:** 2022-04-30

**Authors:** Gianvincenzo Sparacia, Giuseppe Parla, Roberto Cannella, Giuseppe Mamone, Ioannis Petridis, Luigi Maruzzelli, Vincenzina Lo Re, Mona Shahriari, Alberto Iaia, Albert Comelli, Roberto Miraglia, Angelo Luca

**Affiliations:** 1 Department of Radiology, University of Palermo, Palermo, Italy; 2 Radiology Service, Department of Diagnostic and Therapeutic Services, IRCCS-ISMETT (Istituto Mediterraneo per i Trapianti e Terapie ad alta specializzazione), Palermo, Italy; 3 Hepatology Unit, Department for the Treatment and Study of Abdominal Diseases and Abdominal Transplantation, IRCCS-ISMETT (Istituto Mediterraneo per i Trapianti e Terapie ad alta specializzazione), Palermo, Italy; 4 Neurology Service, IRCCS-ISMETT (Istituto Mediterraneo per i Trapianti e Terapie ad alta specializzazione), Palermo, Italy; 5 Department of Neuroradiology, Christiana Care Health System, Newark, DE USA; 6 Ri.Med Foundation, Palermo, Italy

**Keywords:** Hepatic encephalopathy, Cirrhosis, Magnetic resonance imaging, Radiomics, Texture

## Abstract

**Purpose:**

Hepatic encephalopathy (HE) is a potential complication of cirrhosis. Magnetic resonance imaging (MRI) may demonstrate hyperintense T1 signal in the *globi pallidi*. The purpose of this study was to evaluate the performance of MRI-based radiomic features for diagnosing and grading chronic HE in adult patients affected by cirrhosis.

**Methods:**

Adult patients with and without cirrhosis underwent brain MRI with identical imaging protocol on a 3T scanner. Patients without history of chronic liver disease were the control population. HE grading was based on underlying liver disease, severity of clinical manifestation, and number of encephalopathic episodes. Texture analysis was performed on axial T1-weighted images on bilateral lentiform nuclei at the level of the foramina of Monro. Diagnostic performance of texture analysis for the diagnosis and grading of HE was assessed by calculating the area under the receiver operating characteristics (AUROC) with 95% confidence interval (CI).

**Results:**

The final study population consisted of 124 patients, 70 cirrhotic patients, and 54 non-cirrhotic controls. Thirty-eight patients had history of HE with 22 having an HE grade > 1. The radiomic features predicted the presence of HE with an AUROC of 0.82 (95% CI: 0.73, 0.90; *P* < .0001; 82% sensitivity, 66% specificity). Radiomic features predicted grade 1 HE (AUROC 0.75; 95% CI: 0.61, 0.89; *P* < .0001; 94% sensitivity, 60% specificity) and grade ≥ 2 HE (AUROC 0.82; 95% CI: 0.71, 0.93; *P* < .0001, 95% sensitivity, 57% specificity).

**Conclusion:**

In cirrhotic patients, MR radiomic is effective in predicting the presence of chronic HE and in grading its severity.

**Supplementary Information:**

The online version contains supplementary material available at 10.1007/s00234-022-02949-2.

## Introduction

Hepatic encephalopathy (HE) is a cerebral disorder that may occur in the setting of end-stage chronic liver disease [1], presenting with a wide spectrum of neuropsychiatric manifestations. HE is widely considered one of the most serious complications of cirrhosis, representing the initial decompensating event in approximately 20% of these patients and its presence carries a 5-year survival rate of about 20% [2]. HE is the most common cause of protracted hospitalization and readmission in cirrhotic patients and thus poses an enormous financial burden on health care costs [1, 2]. The 5-year incidence of overt HE in cirrhotic patients ranges between 5 and 25%, depending on the underlying cause of chronic liver disease and the presence of additional complications, increasing up to 50% in cirrhotic patient who have undergone transjugular intrahepatic portosystemic shunt (TIPS) for treatment of complications of portal hypertension, including refractory ascites and/or prophylaxis of variceal rebleeding [1–6].

In patients with cirrhosis and HE, a common magnetic resonance imaging (MRI) finding in chronic HE is bilateral symmetric T1 hyperintense signal in the globi pallidi and substantiae nigrae (reported in 80%–90% of patients with chronic liver failure), probably a sequela of chronic accumulation of manganese from failed hepatobiliary excretion. However, this imaging finding does not have a direct or linear correlation with serum ammonia levels and/or patient’s symptoms and it is observed in chronic stages [7]. Furthermore, bilateral T1-weighted bright signal intensity within the globi pallidi may be observed in other disorders that are not necessarily linked to elevated manganese levels [8]. Additional and more complex imaging findings may be seen in cirrhotic patients presenting with an acute encephalopathy, often triggered by a sudden exacerbation of liver dysfunction which leads to an acute elevation of serum ammonia level. These patients’ imaging studies will often demonstrate features specific to acute hyperammonemic encephalopathy superimposed on background imaging findings of chronic HE. Imaging findings of acute HE may include gyriform hyperintense FLAIR signal and corresponding restricted diffusion affecting bilateral cerebral cortices, favoring involvement of the insular cortex and the cingulate gyri and often sparing perirolandic and occipital cortex; these aspects of acute hyperammonemic cortical injury are often superimposed on a background of imaging features of chronic HE, in particular, hyperintense T1 signal in the globi pallidi [7].

Radiomics is emerging as a new quantitative imaging-based method that allows extracting from radiological images some mathematical features that cannot be perceived by human eyes [9–11]. Radiomics and texture-based features reflect the distribution and heterogeneity of signal intensities within a defined region of interest [12]. The extracted features can be used to create clinical decision support systems for prediction of histopathological features, prognosis, and response to therapy, thus explaining the considerable interest that precision medicine has shown in the utility of this emerging methodology [11]. Recent studies in neuroimaging have explored the potential of radiomic and MRI-based texture analysis for the assessment of brain tumors [13, 14], multiple sclerosis lesions [15, 16], and other neurodegenerative disorders [17].

The aim of this study was to evaluate the performance of MRI-based radiomic features for diagnosing the presence of chronic HE and grading its severity, in a population of adult cirrhotic patients.

## Materials and methods

This retrospective study was reviewed and approved by the Institutional Research Review Board of our institution; informed consent form was waived. Informed written consent for magnetic resonance (MR) study was obtained in all subjects who underwent brain MR for clinical purposes.

### Patients population

Eligible patients were identified through a retrospective search of the electronic database of our tertiary transplant center, identifying adult patients who, between October 2018 and February 2020, underwent brain MR exams, all performed with identical imaging protocol.

**Fig. 1:**
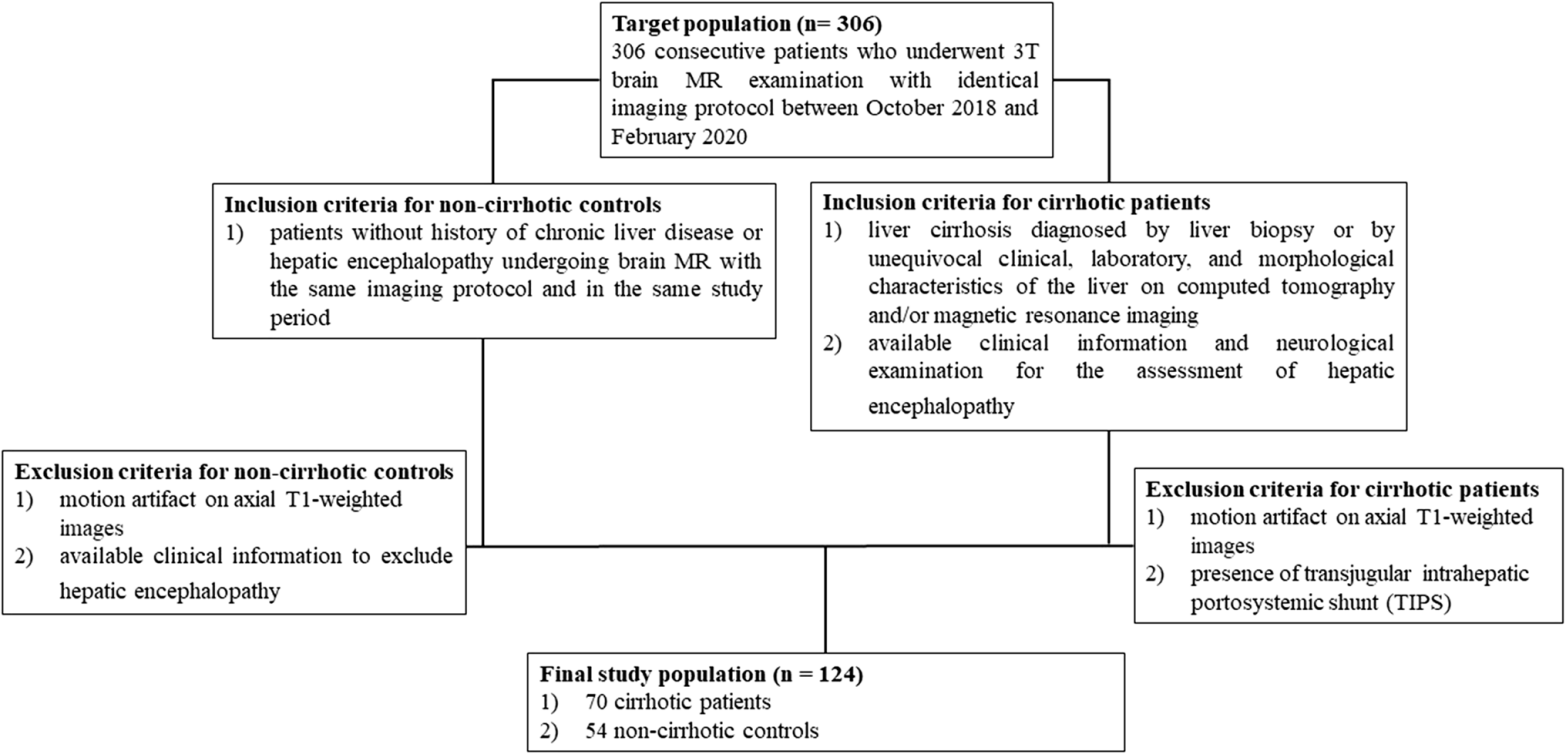
Flowchart shows study enrollment of 306 consecutive patients who underwent 3T brain MR examination with identical imaging protocol between October 2018 and February 2020 with selection of cirrhotic patients and non-cirrhotic controls based on inclusion and exclusion criteria

Cirrhotic patients were selected in the study group if they met the following inclusion criteria (Fig. 1): (1) diagnosis of cirrhosis; (2) brain MRI acquired on a 3T MR scanner using the same scanning protocol; and (3) available clinical information for the assessment of chronic hepatic encephalopathy. Patients were excluded from the study group if they had (1) motion artifact on axial T1-weighted images and (2) presence of transjugular intrahepatic portosystemic shunt (TIPS).

Patients without history of chronic liver disease or chronic hepatic encephalopathy undergoing brain MRI with the same MR scanner and imaging protocol were selected as the control group.

The final study population consisted of 124 patients, 70 cirrhotic patients (74% men, mean age 66 ± 8 years, range 40–86 years), and 54 non-cirrhotic controls (69% men, mean age 62 ± 13 years, range 28–81 years).

The diagnosis of cirrhosis was established by liver biopsy or by unequivocal clinical, laboratory, and morphological characteristics of the liver at computed tomography and/or magnetic resonance imaging. The clinical diagnosis of chronic hepatic encephalopathy was established by a hepatologist (with 15 year of post fellowship experience) who categorized each case according to the number and severity of encephalopathic episodes and the etiology of liver disease [18]. The severity of chronic hepatic encephalopathy was graded according to the Association of the Study of Liver Disease–European Association for the Study of the Liver Practice Guideline using the West Haven criteria (WHC) [1].

### MR imaging acquisition

MR examinations were acquired with a 3T MR scanner (Discovery 750w, General Electric Healthcare, Milwaukee, WI, USA) with a 32-channel dedicated head coil. Axial, sagittal, and coronal non-enhanced and contrast-enhanced (0.1 mmol/kg gadobutrol – Gadovist, Bayer, Germany) fast-spin echo (FSE) T1-weighted (600/20 [TR ms / TE ms]) MR images with a field-of-view (FOV) of 240 mm, matrix 320 × 320, slice thickness 4 mm, intersection gap 1 mm, number of excitations 1 were acquired as a part of standard MR protocol along with axial and sagittal FSE T2-weighted (8000/90 [TR ms/TE ms]) images, and axial fluid attenuated inversion-recovery (FLAIR) (9000/150/2250 [TR ms/TE ms/TI ms]) images.

### Imaging and texture analysis

MRI examinations were evaluated by a neuroradiologist (with 20 years of experience in neuroimaging) in order to further stratify cirrhotic patients according to the presence of hyperintense signal in the basal ganglia on non-contrast T1-weighted images (supplemental material 1).

Texture analysis was performed independently by a second radiologist (6 years of experience in neuroimaging and 3 years of experience with texture analysis), blinded to any patients’ clinical data. For each patient, the axial non-contrast T1-weighted images were anonymized and exported in DICOM format into a dedicated workstation for texture analysis. Images were analyzed in a random order using a freely available texture analysis software (LIFEx, version 5.10, www.lifexsoft.org). Using a single slice, axial T1-weighted image aligned to a similar orientation, the radiologist manually drew a polygonal region of interest (ROI) in bilateral lentiform nuclei at the level of the foramina of Monro. The ROI was placed within the margins of the lentiform nuclei including the largest possible area but carefully avoiding calcifications. None of the subjects presented basal ganglia structural lesion on MRI, apart from incidental mineralization.

A total of 43 texture features (supplemental material 2) were automatically extracted from the software, including first-order features calculated from analysis of the gray level distribution within a defined ROI without considering spatial relations among pixels and second-order features that considered the spatial relationship among pixels. In particular, first-order parameters included conventional (mean, standard deviation) and histogram (reflecting the histogram distribution of pixel intensities). Second-order features included gray level co-occurrence matrix (GLCM) that took into account the arrangements of pairs of pixels to calculate textural indices; gray-level run length matrix (GLRLM); quantified consecutive pixels with the same intensity along specific directions; neighborhood gray-level different matrix (NGLDM), accounting for the difference of gray levels between one pixel and its eight neighbors; and grey-level zone length matrix (GLZLM), providing information on the size of homogeneous zones for each grey-level in different dimensions.

The detailed formula of each texture features has been extensively described in the freely available LifeX online manual [19].

### Computational statistical analysis

Data summarized as continuous variables were expressed as mean and standard deviation (SD), and categorical variables were expressed as numbers and percentages.

Correction for multiple comparisons to achieve a false discovery rate of 5% was performed using the Benjamini–Hochberg procedure [20] and the corrMatching function under MatLab software package version 2019b (The MathWorks Inc., Natick, MA, USA) that allowed one to obtain a maximally normalized Fischer Z-transformation cross-correlation texture analysis parameters with each other in both directions [21]. This procedure allows to select the most discriminative texture features, avoiding repetition of multiple features with high similarity or with incidental statistical significance. Correlation matrix was used in order to identify the combination of the most discriminative features able to differentiate each of the following condition: (1) cirrhotic patients versus controls; (2) the presence of HE in cirrhotic patients; (3) the grade of HE in cirrhotic patients (grade 1 vs grade > 2); and (4) the value of the T1-weighted hyperintensity in the globi pallidi in predicting the presence of HE in cirrhotic patients.

Multiparametric logistic regression models and classifiers were tested using as the dependent variable the presence of each condition, as covariates the combination of the most discriminative texture analysis features previously selected. The area under the receiver operating characteristics (AUROC) with 95% confidence interval (CI) and *p* value were calculated. Cutoff values for the identification of each condition with the combined features were calculated by means of the highest Youden index with the relative sensitivity, specificity, accuracy, positive predictive value (PPV), and negative predictive value (NPV) values.

Statistical analysis was conducted using SPSS Statistics software package version 25 (SPSS, Chicago, USA) and the ROC curve fitting function version 2 under MatLab [22].

## Results

### Patients’ characteristics

Baseline characteristics of the patients included are summarized in Table 1

**Table 1:** Characteristics of included patients

Characteristics	Cirrhotic (*n* = 70)	Controls (*n* = 54)
Age (years) mean ± SD (range)	66.2 ± 8.4 (40–86)	62.4 ± 13 (28–81)
Sex		
Men Women	52 (74%) 18 (26%)	37 (69%) 17 (31%)
Etiology of the cirrhosis		
Viral NASH Alcohol Others	41 (59%) 11 (16%) 10 (14%) 8 (11%)	- - - -
Hepatic encephalopathy	38 (54%)	0 (0)
HE Grade		
Grade 1 Grade 2 Grade 3 Grade 4	16 (23%) 16 (23%) 4 (6%) 2 (3%)	- - - -
Type of HE		
Episodic Recurrent Persistent	14 (20%) 11 (16%) 13 (19%)	- - -
MELD, mean ± SD (range)	14.0 ± 5.6 (6–40)	-

Abbreviations: *SD* standard deviation, *HE* hepatic encephalopathy, *NASH* non-alcoholic fatty liver disease, *MELD* Model for End-Stage Liver Disease

Clinical indications for brain MRI in cirrhotic patients included pre-transplant evaluations in 52 (74%) patients, assessment of changes in the SI in the *globi pallidi* related to chronic HE in 8 (12%) patients, and other neurological manifestations in 10 patients (14%). At clinical examination, chronic HE was confirmed in 38 (54%) of cirrhotic patients. HE was classified as grade 1 in 16 (23%) patients, while 22 (32%) had HE grade ≥ 2. Episodic, recurrent, and persistent HE was present in 14 (20%), 11 (16%), and 13 (19%) patients, respectively.

Clinical indications for brain MRI in patients without history of chronic liver disease or hepatic encephalopathy included memory disturbance in 6 (11%) patients, migraine in 11 (20%) patients, chronic hypoxic-ischemic encephalopathy in 31 (57%) patients, optic neuropathy in 2 (4%) patients, and cortical laminar necrosis in 4 (8%) patients.

### Diagnostic performance of T1-weighted hyperintensity in basal ganglia

**Fig. 2:**
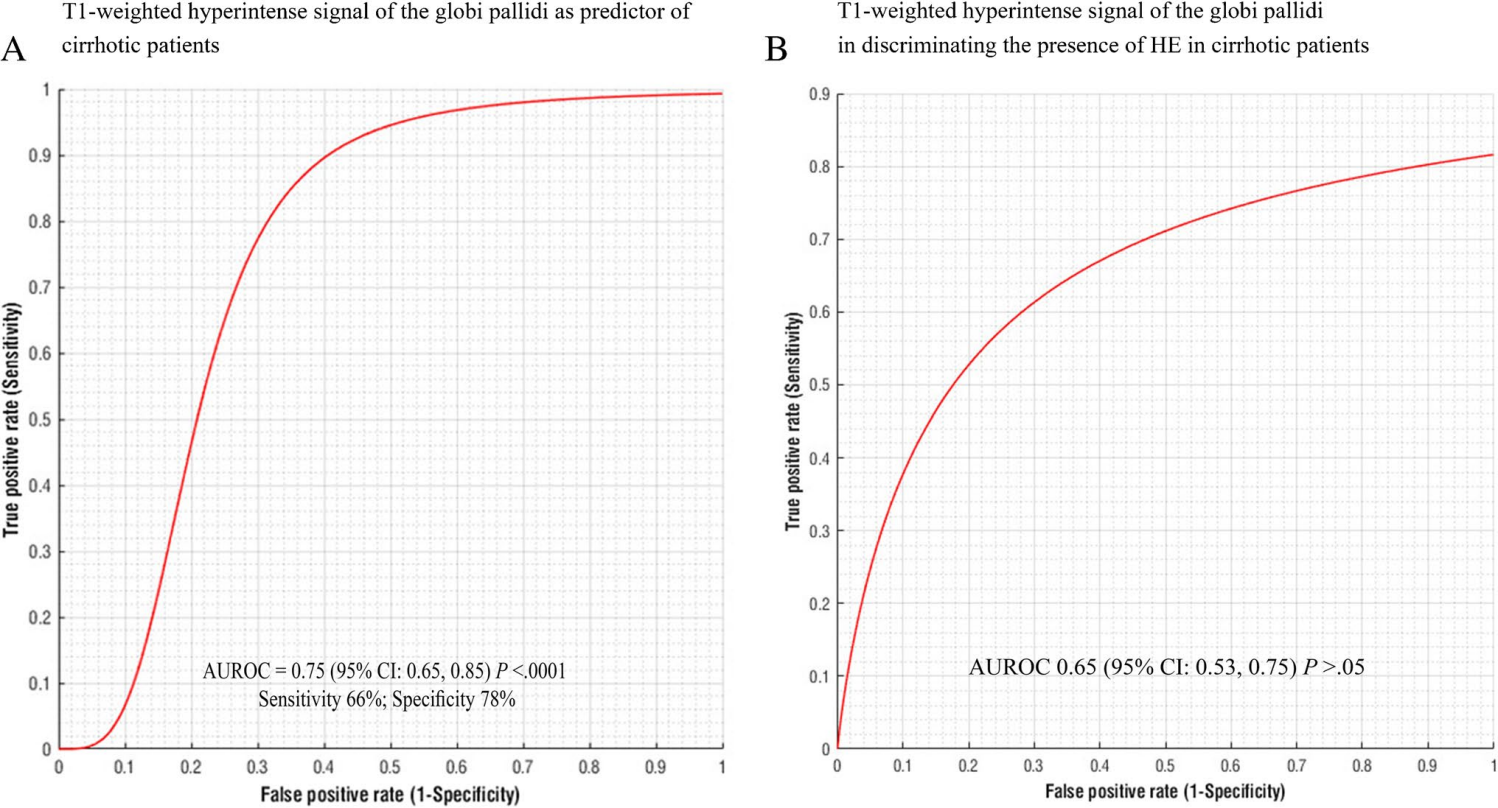
(**a**) Graph representing the AUROC for the T1-weighted hyperintense signal of the globi pallidi as predictor of cirrhotic patients. The T1-weighted hyperintense signal of the globi pallidi was statistically significant discriminator for identification of cirrhotic patients (AUROC = 0.75; 95% CI: 0.65, 0.85; *P* < .0001). (**b**) Graph represents the AUROC for the T1-weighted hyperintense signal of the globi pallidi in discriminating the presence of chronic HE in cirrhotic patients. The T1-weighted hyperintense signal of the globi pallidi was not statistically significant discriminator for the presence of chronic HE in cirrhotic patients (AUROC 0.65; 95% CI: 0.53, 0.75; *P* > .05). AUROC, area under the receiver operator characteristics curve; CI, confidence intervals

Hyperintense signal in the basal ganglia (*globus pallidus*) on T1-weighted imaging was qualitatively reported in 34 (49%) patients with cirrhosis. The hyperintense signal on T1-weighted imaging demonstrated a fair diagnostic performance for the identification of cirrhotic patients with an AUROC of 0.75 (95% CI: 0.65, 0.85; *P* < 0.0001; sensitivity 66%; specificity 78%) (Fig. 2a). However, the hyperintense signal on T1-weighted imaging was not a statistically significant discriminator for the presence of HE in cirrhotic patients (AUROC 0.65; 95% CI: 0.53, 0.75; *P* > 0.05) (Fig. 2b).

### Diagnostic performance of texture-based models

Eight uncorrelated texture-based features (GLRLM_GLNU, GLRLM_LGRE, GLRLM_SRHGE, GLRLM_SRLGE, GLZLM_GLNU, GLZLM_LGZE, GLZLM_SZLGE, GLZLM_ZP) were selected with the method described which avoids repetition of multiple features with high similarity or with incidental statistical significance. The combination of these eight texture-based features were significant predictors of cirrhosis and hepatic encephalopathy.

**Fig. 3:**
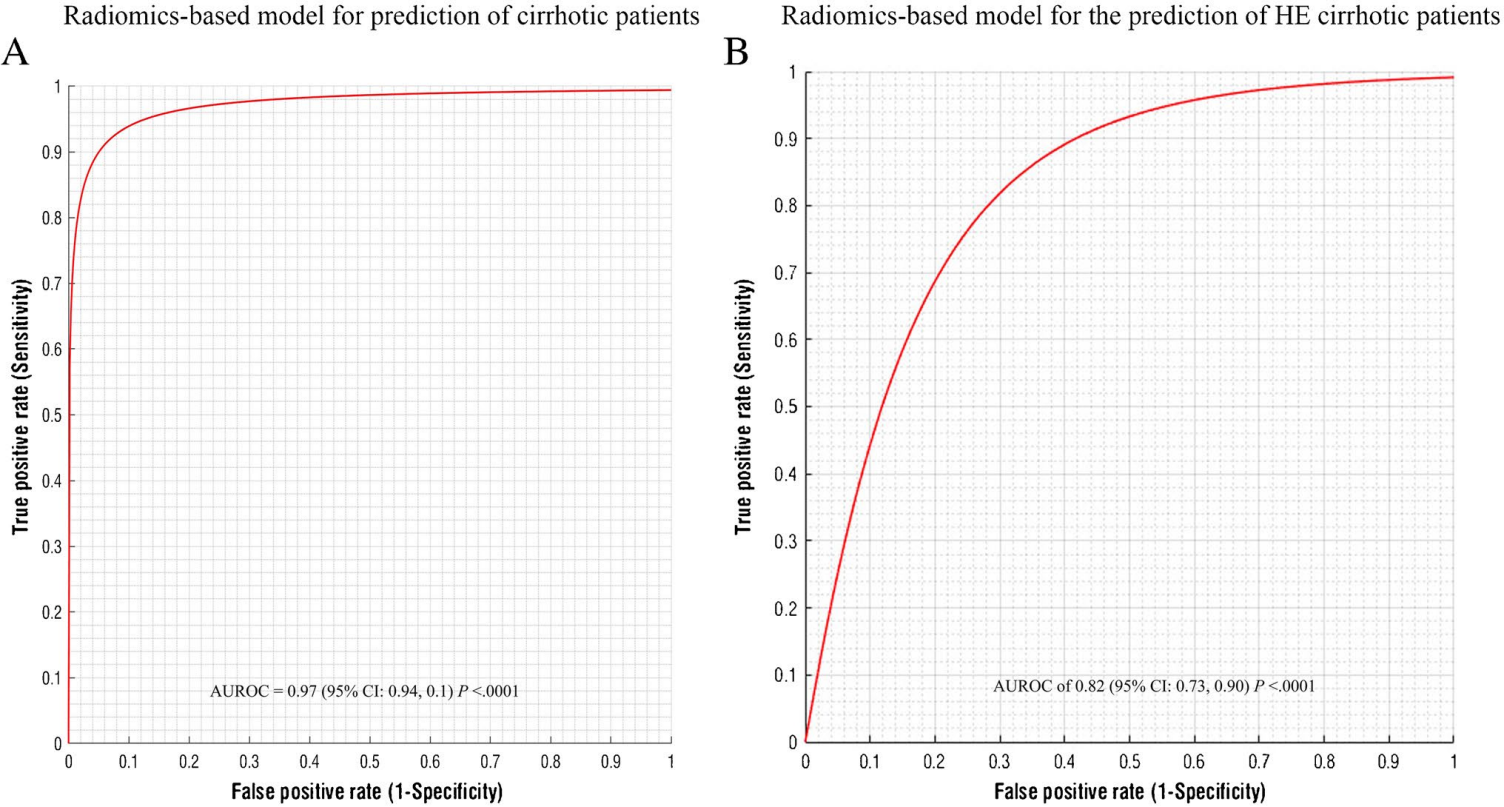
(**a**) Graph representing the AUROC for radiomics-based model for prediction of cirrhotic patients having a statistically significant high prediction value (AUROC = 0.97; 95% CI: 0.94, 0.1, *P* < .0001). (**b**) Graph representing the AUROC for radiomics-based model for the prediction of chronic HE cirrhotic patients. The radiomic-based model showed a significant prediction value in discriminating the presence of chronic HE in cirrhotic patients (AUROC of 0.82; 95% CI: 0.73, 0.90; *P* < .0001). AUROC, area under the receiver operator characteristics curve; CI, confidence intervals

Performance of multiparametric logistic regression models is reported in Table 2. Radiomic-based model demonstrated an excellent diagnostic performance for the identification of cirrhotic patients with an AUROC of 0.97 (95% CI: 0.94, 0.1, *P* < 0.0001) (Fig. 3a). A cutoff value > 0.5 selected by means of the highest Youden index had a sensitivity of 94% and a specificity of 91% for the prediction of HE. The radiomic model provided a good performance for the diagnosis of HE in cirrhotic patients with an AUROC of 0.82 (95% CI: 0.73, 0.90; *P* < 0.0001) (Fig. 3b). A cutoff value > 0.37 had a sensitivity of 82% and a specificity of 66% for the prediction of HE.

**Table 2:** Diagnostic performance of texture-based model for the assessment of cirrhosis, hepatic encephalopathy (HE), and the grade of HE

Outcome prediction	AUROC (95%, CI)	*P* value	Optimal cutoff	Sensitivity	Specificity	Accuracy	PPV	NPV
Cirrhosis	0.97 (0.94, 0.1)	< .0001	> .5	94%	91%	93%	99%	45%
HE (any grade)	0.82 (0.73, 0.90)	< .0001	> .37	82%	66%	71%	96%	26%
HE = 1	0.75 (0.61, 0.90)	< .0001	> .13	94%	60%	63%	90%	70%
HE ≥ 2	0.83 (0.71, 0.93)	< .0001	> .08	95%	57%	64%	93%	67%
MELD score	0.94 (0.9, 0.1)	< .0001	> 9.7	91%	81%	87%	99%	37%

Abbreviations: *AUROC* area under the receiver operator characteristics curve, *CI* confidence intervals, *PPV* positive predictive value, *NPV* negative predictive value. Optimal cut-off is based on Youden’s index

**Fig. 4:**
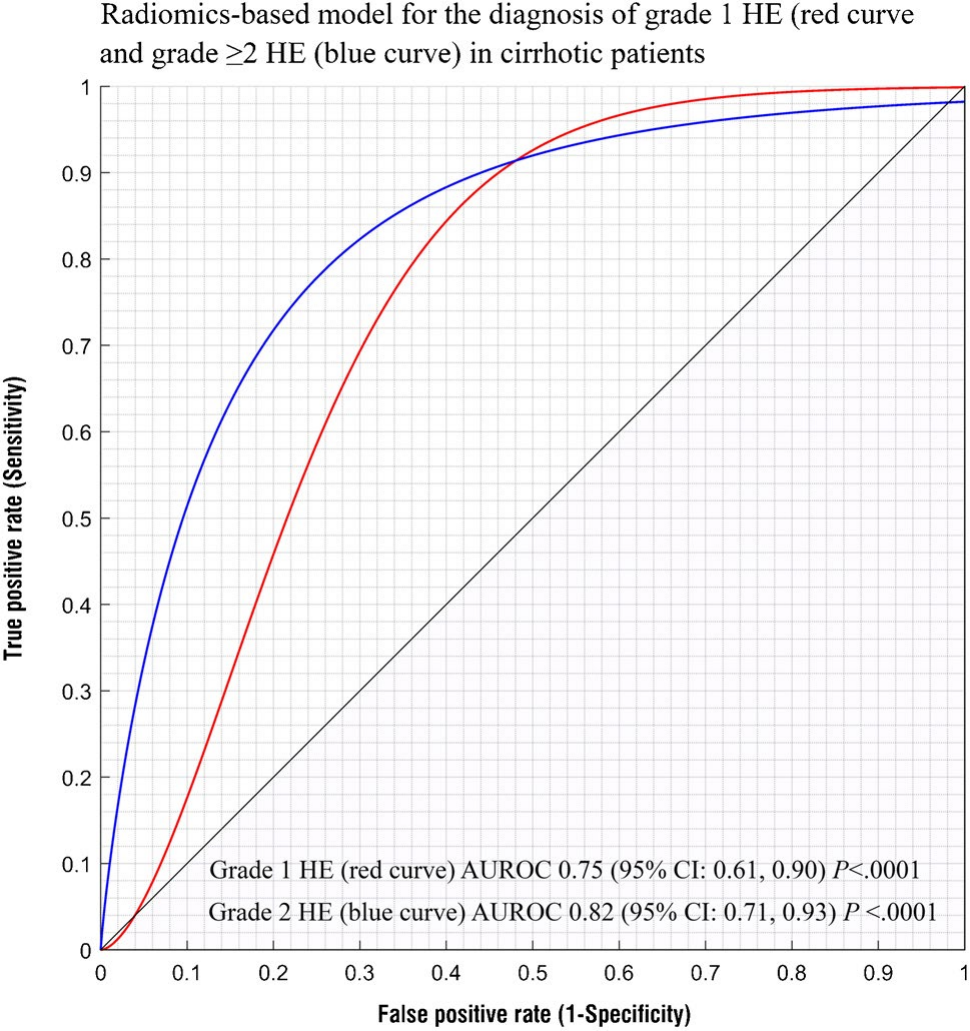
Graph representing the AUROC of radiomics-based model for the diagnosis of grade 1 chronic HE (red curve) and grade ≥ 2 chronic HE (blue curve) in cirrhotic patients. The performance of radiomics-based model for the diagnosis of grade 1 chronic HE had an AUROC of 0.75 (95% CI: 0.61, 0.90; *P* < .0001) and an AUROC of 0.82 (95% CI: 0.71, 0.93; *P* < .0001) for grade ≥ 2 chronic HE. Radiomics-based model was significantly predictive for both grade 1 chronic HE and grade ≥ 2 chronic HE in cirrhotic patients. AUROC, area under the receiver operator characteristics curve; CI, confidence intervals; HE, hepatic encephalopathy

When considering the HE grades, the performance of radiomic-based model was fair for the diagnosis of grade 1 HE (AUROC 0.75; 95% CI: 0.61, 0.90; *P* < 0.0001) and good for grade ≥ 2 HE (AUROC 0.82; 95% CI: 0.71, 0.93; *P* < 0.0001) (Fig. 4). The sensitivity and specificity for the diagnosis of grade 1 HE were 94% and 60%, respectively, using the optimal cutoff value > 0.13; while for the diagnosis of grade ≥ 2 HE, the sensitivity and specificity were 95% and 57% respectively using the optimal cutoff value > 0.08.

**Fig. 5:**
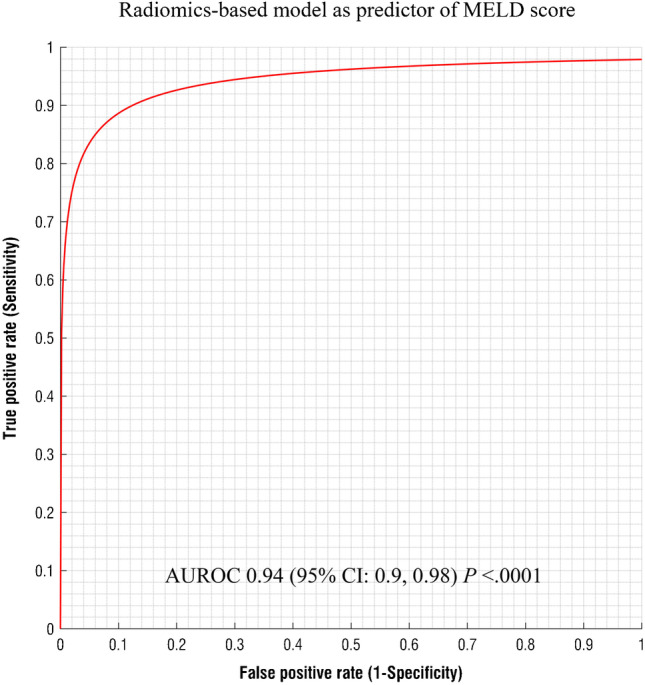
Graph representing the AUROC of radiomics-based model as predictor of MELD score. The radiomics-based model provided significant performance as predictor of MELD score in cirrhotic patients with an AUROC of 0.94 (95% CI: 0.9, 0.98; *P* < .0001). AUROC, area under the receiver operator characteristics curve; CI, confidence intervals; MELD, Model for End-Stage Liver Disease

When considering the patients Model for End-Stage Liver Disease (MELD) score, the radiomic model performed well with an AUROC 0.94 (95% CI: 0.9, 0.1; *P* < 0.0001) (Fig. 5) with a sensitivity of 91% and a specificity of 81% when using the optimal cutoff of > 9.7.

## Discussion

Our study demonstrates that MRI-based texture-analysis radiomic models may provide clinically valuable information for the diagnosis and staging of chronic HE in cirrhotic patients. The combination of texture features extracted on T1-weighted imaging was highly accurate in predicting the presence of cirrhosis (AUROC of 0.97) and moderately accurate for predicting the presence of chronic HE (AUROC of 0.82). To the best of our knowledge, this is the first study investigating the potential use of brain MR radiomics for predicting the presence of HE in cirrhotic patients.

The pathological basis of HE in cirrhosis are not fully understood. In the setting of significant liver dysfunction, increased hepatic resistance to the normal enterohepatic circulation forces gut-derived toxins, including ammonia and inflammatory cytokines, into the systemic circulation via spontaneous portosystemic shunts. Subsequently, ammonia is taken up by the brain, a process that has been shown to be associated with edematous changes affecting astrocytes and neurons. Chronic cerebral ammonia exposure leads to irreversible structural changes in astrocytes [5]. The mechanism for astrocyte swelling in acute liver failure remains uncertain but is likely to include excessive generation of osmolytes, mainly glutamine, within the astrocytes as a result of ammonia detoxification through the action of glutamine synthetase. Additionally, abnormalities in intracellular pH and membrane potential can disrupt ion homeostasis and lead to cell swelling [23]. The relatively more rapid evolution of the HE in fulminant hepatic failure, compared to the chronic liver failure, may explain why the homeostatic compensatory metabolic changes are not allowed to counteract the osmotic unbalance induced by intra-astrocytic glutamine accumulation, while in chronic liver failure there is enough time for activation of effective compensatory mechanisms of cellular adaptation to the osmotic change [24]. In patients with cirrhosis subjected to ammonia load, this mechanism of osmolar adaptation is clearly reflected in proton MR spectroscopy studies, which consistently show increases in the glutamine/glutamate signal intensity accompanied by myo-inositol depletion and decreases in the choline signal intensity [25]. This osmoregulatory mechanism is activated after liver failure and accounts for the protection against massive edema in chronic liver failure [26]. Other factors including inflammation, oxidative stress, increased bile acids, and lactate contribute to the progression and severity of HE [6].

In patients with cirrhosis and HE, a common magnetic resonance imaging (MRI) finding in chronic HE is bilateral symmetric T1 hyperintense signal in the globi pallidi and substantiae nigrae (reported in 80%–90% of patients with chronic liver failure), probably a sequela of chronic accumulation of manganese from failed hepatobiliary excretion then transferred to the brain through the blood–brain barrier by several transport systems. This increase in brain manganese has a neurotoxic effect, inducing selective neuronal loss in basal ganglia structures and reactive gliosis. These effects are more prominent in the globus pallidus; the substantia nigra reticulata; and, to a lesser extent, the striatum, with sparing of the substantia nigra pars compacta neurons and striatal dopamine [26]. However, T1 hyperintense signal-intensity alterations in the globi pallidi are not closely linked to the presence of HE. Patients with cirrhosis and no clinical signs of HE can also show signal-intensity alterations, whereas others with HE may present slight signal-intensity alterations [27]. Moreover, studies have shown quick regression of HE after liver transplantation, whereas T1 signal intensity abnormalities need up to 1 year to resolve [28]. This clinical-MR imaging discrepancy may explain why the T1 high signal intensity in the globi pallidi cannot be used as a quantitative measure of tissue manganese deposition because it represents solely a semiquantitative measurement of abnormal manganese deposition. Thus, it is possible that manganese accumulation participates in the pathogenesis of HE only after reaching a certain degree, which may not be clearly identified by MR imaging.

Hepatic encephalopathy classification relies on 2 scales: the International Society for Hepatic Encephalopathy and Nitrogen Metabolism (ISHEN) and the West Haven Criteria (WHC) [1]. The ISHEN makes a distinction between covert HE and overt HE. Patients with covert HE shows little to no clinical symptoms and usually do not require hospitalization, while overt HE is characterized by temporal and spatial disorientations or the presence of asterixis and usually require hospitalization [3]. The WHC classification, used to define the grade of HE in this study, includes 6 stages: unimpaired, minimal, and grades I through IV HE. From a clinical standpoint, differentiation between minimal HE and grade I HE is intangible while grades II through IV describe overt HE, and range in severity from asterixis in grade II, to coma in grade IV [1].

We speculated that radiomics of globi pallidi may be able to capture the regional heterogeneity in the affected brain areas and, therefore, provide good-to-excellent performances for the identification of cirrhotic patients and chronic HE.

In our study, the radiomic models provided higher sensitivity for the prediction of cirrhosis and chronic HE (sensitivities of 94% and 82%, respectively) compared to the hyperintensity on T1-weighted images (sensitivity of 66% for prediction of cirrhosis status), which is classically considered the MR imaging features of chronic HE in cirrhotic patients.

The radiomic models provided a fair-to-good performance for the diagnosis of grade 1 HE (AUROC of 0.75) and grade ≥ 2 HE (AUROC 0.83). The cutoff values provided by radiomic models have demonstrated high sensitivity for the diagnosis of HE grades. In particular, a cutoff > 0.13 had a sensitivity of 94% for grade 1 HE, while a cutoff > 0.08 had a sensitivity of 95% for grade ≥ 2 HE, while the specificity remained limited (60–57%, respectively).

In this setting, the radiomic-based models may be used in clinical practice to maximize the sensitivity for the MRI diagnosis of chronic HE especially in cirrhotic patients undergoing MR imaging for surveillance and with minimal neurological symptoms, which could be further confirmed at clinical evaluation. The possibility of discriminating between cirrhotic and non-cirrhotic patients through brain MR radiomic features could assist in establishing the underlying etiology of chronic liver disease in patients who undergo brain MRI for other causes.

The preliminary findings of our study may stimulate additional research projects including, for instance, the search for radiomic features that may be predictive of post TIPS HE, in order to better stratify patients before TIPS procedure. Post-TIPS HE is the major drawback of TIPS, with a reported incidence of 30–50%; while symptoms are mild in most patients, they can be severe in some post TIPS patients, even requiring hospitalization [16]. Post TIPS HE poses an enormous financial burden for healthcare systems; in a large recent study, performed in a cohort of about 28,000 hospitalized patients who underwent TIPS procedure, the rate of 30-day readmissions was 28%, and the most common reason for readmissions was related to HE, with or without coma, in at least one-third of the patients [17]. Several clinical factors such as older age, history of HE, higher Child–Pugh/MELD score, poor nutritional status, and low serum sodium, are associated to post TIPS HE [16]; yet, it remains challenging to correctly identify patients at risks prior to performing TIPS. The potential for developing post TIPS HE is a leading cause for hepatologists’ hesitation in recommending TIPS, especially in patients with refractory ascites in whom post TIPS survival is lower than in patients with variceal bleeding as their main indication for a TIPS. The potential benefit of adding brain radiomic features as an additional tool for optimized patients’ selection for TIPS could theoretically lead to lower incidence of post TIPS HE. Another potential future study might involve applying this methodology to evaluate its performance in the setting of acute encephalopathies. In particular, one could evaluate the performance of this technique in detecting the presence of underlying HE in patients presenting with an acute, non-specific encephalopathic status, to establish its ability to separate the acutely ill patients who are affected by chronic HE from those without underlying HE.

Our study presents some limitations that must be acknowledged. First, this was a retrospective study with a limited number of cirrhotic patients and controls and we did not investigate the inter-reader agreement for texture features evaluation. Second, all subjects were imaged using a 3T MR scanner. Therefore, these results may be not generalizable to 1.5 T MR scanners, which are still frequently employed for the clinical assessment of cirrhotic patients. Additionally, radiomic model was based on only axial non-contrast T1-weighted images. We did not explore the performance of radiomics in other sequences such as FLAIR or DWI, which may demonstrate more subtle changes related to chronic HE. Given the findings of our study, radiomics applied to sequence other than T1 alone, including FLAIR and DWI, might make for a very interesting subject of future research. Finally, our sample of cirrhotic patients had a mean MELD score of 14 indicating a severe liver disease; these results should therefore be confirmed in patients with less advanced liver disease.

Further studies are needed to validate the proposed results in large multicentric cohorts including control groups undergoing MRI for different clinical indications and by evaluating radiomic changes related to chronic HE in other sequences such as FLAIR or DWI.

In conclusion, MRI-based texture analysis proved to be highly accurate in predicting the presence of cirrhosis and chronic hepatic encephalopathy and effective in correctly staging HE grade in cirrhotic patients.

## Supplementary Information

Below is the link to the electronic supplementary material.

Supplementary file1 (DOCX 14 KB)

Supplementary file2 (DOCX 301 KB)

## Data Availability

Yes, if requested (by GitHub).

## Abbreviations

AUROC: Area under the ROC curve
FSE: Fast-spin echo
FOV: Field-of-view
MELD: Model for End-Stage Liver Disease
MR: Magnetic resonance
MRI: Magnetic resonance imaging
ROI: Region of interest
ROC: Receiver operating characteristics
SD: Standard deviation
TA: Texture analysis
