# Supplementary material for: Brain magnetic resonance imaging radiomics features associated with hepatic encephalopathy in adult cirrhotic patients
Source: Neuroradiology. 2022 Apr 30;64(10):1969–78. doi: 10.1007/s00234-022-02949-2 (PMC9474333; doi:10.1007/s00234-022-02949-2)
Supplement: Supplementary file 1 — Supplementary file1 (DOCX 14 KB) [file 234_2022_2949_MOESM1_ESM.docx]

**Supplemental material 2:** Texture features extracted using LifeX software.

| First Order Texture Features | | | |
| --- | --- | --- | --- |
| Conventional  CONVENTIONAL_min CONVENTIONAL_mean  CONVENTIONAL_std  CONVENTIONAL_max  CONVENTIONAL_Q1  CONVENTIONAL_Q2  CONVENTIONAL_Q3 | | **Histogram**  HISTO_Skewness  HISTO_Kurtosis  HISTO_Entropy_log2  HISTO_Entropy_log10  HISTO_Energy | |
| Second order Texture Features | | | |
| GLCM  GLCM_Homogeneity  GLCM_Energy  GLCM_Contrast  GLCM_Correlation  GLCM_Entropy  GLCM_Dissimilarity | **GLRLM**  GLRLM_SRE  GLRLM_LRE  GLRLM_LGRE  GLRLM_HGRE  GLRLM_SRLGE  GLRLM_SRHGE  GLRLM_LRLGE  GLRLM_LRHGE  GLRLM_GLNU  GLRLM_RLNU  GLRLM_RP | **NGLDM**  NGLDM_Coarseness  NGLDM_Contrast  NGLDM_Busyness | **GLZLM**  GLZLM_SZE  GLZLM_LZE  GLZLM_LGZE  GLZLM_HGZE  GLZLM_SZLGE  GLZLM_SZHGE  GLZLM_LZLGE  GLZLM_LZHGE  GLZLM_GLNU  GLZLM_ZLNU  GLZLM_ZP |

**Abbreviations:**

**GLCM** Gray-level co-occurrence matrix

**GLRLM** Gray-level run length matrix

**GLRLM_SRE** Short-Run Emphasis

**GLRLM_LRE** Long-Run Emphasis

**GLRLM_LGRE** Low Gray-level Run Emphasis

**GLRLM_HGRE** High Gray-level Run Emphasis

**GLRLM_SRLGE** Short-Run Low Gray-level Emphasis

**GLRLM_SRHGE** Short-Run High Gray-level Emphasis

**GLRLM_LRLGE** Long-Run Low Gray-level Emphasis

**GLRLM_LRHGE** Long-Run High Gray-level Emphasis

**GLRLM_GLNUr** Gray-Level Non-Uniformity for run

**GLRLM_RLNU** Run Length Non-Uniformity

**GLRLM_RP** Run Percentage

**GLZLM** Gray-level zone length matrix

**GLZLM_SZE** Short-Zone Emphasis

**GLZLM_LZE** Long-Zone Emphasis

**GLZLM_LGZE** Low Gray-level Zone Emphasis

**GLZLM_HGZE** High Gray-level Zone Emphasis

**GLZLM_LZLGE** Long-Zone Low Gray-level Emphasis

**GLZLM_LZHGE** Long-Zone High Gray-level Emphasis

**GLZLM_GLNUz** Gray-Level Non-Uniformity for zone

**GLZLM_ZLNU** Zone Length Non-Uniformity

**GLZLM_ZP** Zone Percentage

**NGLDM** Neighborhood gray-level different matrix

**LifeX online manual** <https://www.lifexsoft.org/images/phocagallery/documentation/ProtocolTexture/UserGuide/TextureUserGuide.pdf>
