# Supplementary material for: Brain magnetic resonance imaging radiomics features associated with hepatic encephalopathy in adult cirrhotic patients
Source: Neuroradiology. 2022 Apr 30;64(10):1969–78. doi: 10.1007/s00234-022-02949-2 (PMC9474333; doi:10.1007/s00234-022-02949-2)
Supplement: Supplementary file 2 — Supplementary file2 (DOCX 301 KB) [file 234_2022_2949_MOESM2_ESM.docx]

**Supplemental material 2:**

**
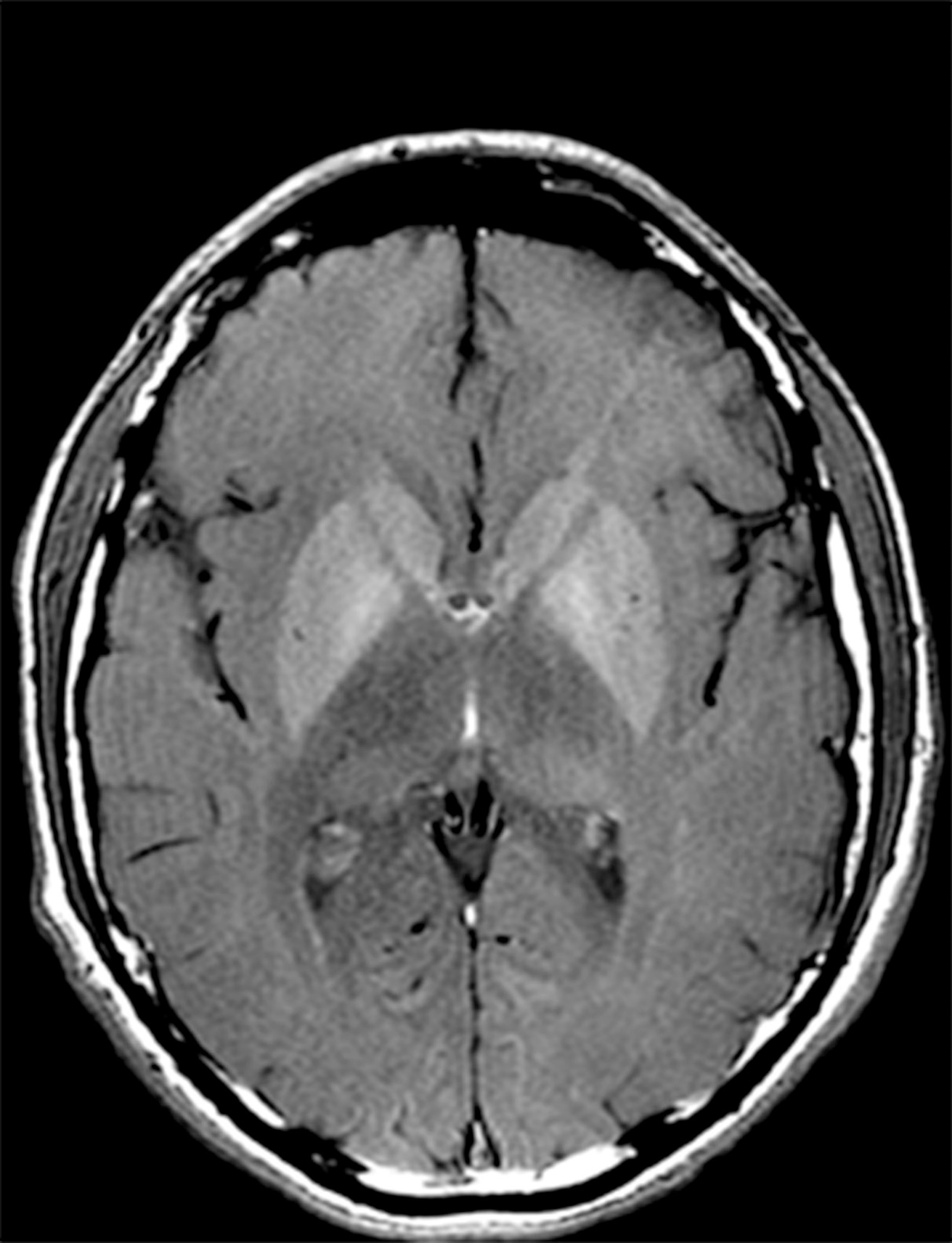
**

Brain non-contrast axial T1-weighted MR image shows T1 hyperintense signal in the basal ganglia in a sixty-five-year-old man with alcoholic related cirrhosis.
